# High‐intensity exercise training improves perioperative risk stratification in the high‐risk patient

**DOI:** 10.14814/phy2.14409

**Published:** 2020-05-06

**Authors:** George A. Rose, Michael J. Adamson, Richard G. Davies, Ian R. Appadurai, Damian M. Bailey

**Affiliations:** ^1^ Faculty of Life Sciences and Education University of South Wales Pontypridd UK; ^2^ Department of Anaesthetics University Hospital of Wales Cardiff UK

**Keywords:** cardiopulmonary exercise test, exercise training, risk assessment

## Abstract

Exercise prehabilitation prior to major surgery can improve cardiorespiratory fitness (CRF) and clinical outcome. However, in patients deemed “high‐‐risk” for surgery, the feasibility, optimum training modality and its intensity, duration, and frequency are yet to be defined. We assessed the cardiorespiratory fitness of a 70‐year‐old female patient requiring major thoraco‐abdominal surgery for reconstruction of her esophagus. Cardiopulmonary exercise testing (CPET) on a cycle ergometer was used to determine CRF. A baseline CPET confirmed poor CRF and placed her in a high surgical risk group. This was followed by 16 weeks of unsupervised, home‐based, moderate‐intensity steady‐state (MISS) training followed by 10 weeks of high‐intensity interval training (HIIT) under the combined supervision of an exercise physiologist and clinician in hospital. Following MISS training, CPET metrics failed to improve: peak oxygen uptake decreased (14.7–13.7 ml O_2_·kg^−1^·min^−1^; −7%) together with peak power (73–70 W; −4%) and anaerobic threshold (AT) increased (7.8–8.3 ml O_2_·kg^−1^·min^−1^; +6%). However, HIIT resulted in impressive improvement in CRF. Peak oxygen uptake (13.7–18.6 ml O_2_·kg^−1^·min^−1^; +36%), AT (8.3–10.5 ml O_2_·kg^−1^·min^−1^; +27%), peak power (70–102 W; +46%), minute ventilation (35.8–57.7 L·min^−1^; +61%), and peak heart rate (100–133 b·min^−1^; +33%) all increased. Ventilatory equivalents for carbon dioxide at AT (${\dot{V}}_{E}$/$\dot{V}$CO_2_‐AT) improved (30–28; −7%). The improvement in CRF resulted in surgical reclassification from high to low risk. In conclusion, preoperative HIIT training can confer a marked improvement in CRF in an elderly surgical patient and is associated with a corresponding reduction in perioperative risk.

## Graphical abstract

A 70-year-old female patient requiring major thoraco-abdominal surgery for reconstruction of her esophagus underwent cardiopulmonary exercise testing, which identified poor cardiorespiratory fitness (CRF) and placed her in a high surgical risk group. Ten weeks of supervised preoperative high-intensity interval training (HIIT) enabled impressive improvement in CRF (36% increase in peak oxygen uptake). The improvement in CRF resulted in surgical reclassification from high to low risk.

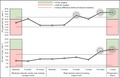

## 1 INTRODUCTION

Poor cardiorespiratory fitness (CRF) is associated with an increased risk of adverse perioperative outcomes including major morbidity, mortality, increased length of stay in hospital (Moran et al., 2016) and reduced health‐related quality of life (Tew, Ayyash, Durrand, & Danjoux, 2018) following major surgery. The American Heart Association guidelines (2014) recommend functional assessment for evaluating peri‐operative risk (Fleisher et al., 2014). Cardiopulmonary exercise testing (CPET) is used to objectively measure functional capacity and can identify the causes of exercise limitation. CPET can evaluate chronic comorbidities and allow identification of new pathology that requires treatment or optimization. These data can be used to facilitate shared decision making, to allow appropriate utilization of postoperative critical care and to direct prehabilitation programs. Approximately 30,000 preoperative CPET are conducted in the UK each year to assess patient risk and plan care (Reeves et al., 2018). With the rapid uptake of CPET, an international Perioperative Exercise Testing and Training Society has been established to promote the highest standards of care for patients undergoing exercise testing, training, or both in the perioperative setting (Levett et al., 2018). There is increasing evidence that preoperative exercise training can improve CRF (West et al., 2015) by creating improved physiological reserve to deal with the stress response to surgery. Typically, studies recruit by convenience with younger and physically active patients more likely to participate. Thus, the feasibility and efficacy of exercise interventions in “unfit” patients deemed high risk for surgery is not adequately addressed and warrants further investigation.

It is well established that moderate intensity steady‐state (MISS) exercise can improve CRF reducing the risk of cardiovascular disease and all‐cause mortality across the human aging continuum (Blair, Kohl, & Paffenbarger, 1989). However, the optimal modality, frequency, and duration remain a constant source of debate. Furthermore, clinical urgency and time demands may be potential barriers to participation (Reichert, Barros, Domingues, & Hallal, 2007). As a consequence, attention has since turned to an alternative exercise modality, high‐intensity interval training (HIIT), given its capacity to further potentiate metabolic, cardiopulmonary, and systemic vascular adaptation with the added attraction of reduced exercise duration even in patients who are deemed “high risk” (Gibala et al., 2006). With this in mind, we describe a clinical case study to highlight the feasibility and potential benefits of HIIT in a high‐risk patient requiring esophageal reconstruction in an attempt to improve postoperative outcome.

## 2 METHODS

### 2.1 Ethics approval

The Cardiff and Vale University Health Board Ethics Committee was informed and formal approval was deemed unnecessary as this was part of the proposed preoperative optimization strategy. The patient provided written informed consent and all procedures adhered to guidelines set forth in the Declaration of Helsinki.

### 2.2 Patient

A 70‐year‐old Caucasian female with a body mass of 24 kg·m^−2^, hemoglobin of 12 g·dl^−1^ and normal renal and liver function underwent transhiatal esophagectomy for esophageal cancer but developed postoperative ischemia of the gastric conduit. Following a problematic course on critical care she required further emergency surgery and was left with a pharyngostomy and a feeding jejunostomy. The patient attended our anesthetic preoperative clinic for assessment of CRF for colonic interposition to restore gastrointestinal tract continuity. Her medical history included myocardial infarction, coronary artery bypass surgery, hypertension, pulmonary embolism, and a right hemi‐colectomy for cecal cancer. Her drug treatment included apixaban, ramipril, bisoprolol, and atorvastatin. She denied symptoms of angina and had a good self‐reported tolerance to physical activity despite a 30‐pack year smoking history.

### 2.3 Design

#### 2.3.1 Exercise interventions

Following initial CPET, she was stratified as high risk for surgical intervention and the patient attempted to improve her functional capacity with unsupervised training at home using a treadmill walking for 20 min, three times per week (MISS training).

A second CPET, 8 weeks later, demonstrated no difference to her risk stratification and she agreed to train further using a home fitness video three times per week. Despite being well motivated, the patient's own efforts failed to improve her CPET metrics. This led to further detailed discussion of perioperative risk and adequate preoperative preparation, and she agreed to undertake a 10‐week HIIT exercise program jointly supervised by an exercise physiologist and clinician.

HIIT consisted of three exercise sessions per week on a cycle ergometer, each of 40 min duration. Sessions comprised six, 2‐min bouts of heavy exercise (50% difference between power output at peak exercise and anaerobic threshold [AT]) interspersed with 3 min of moderate exercise (80% power at AT) based on previous research by West et al. (2015). Heart rate (3‐lead electrocardiogram), blood pressure, and oxygen saturations by finger pulse oximetry were monitored during exercise. A CPET was conducted approximately every 2 weeks and HIIT intensity adjusted accordingly. A final CPET was performed 2 weeks prior to surgery to assess changes in functional capacity following HIIT.

### 2.4 Measurements

#### 2.4.1 CPET

Objective assessment of functional capacity was performed using CPET. All CPETs were conducted to volitional fatigue using a MedGraphics Ultima metabolic cart (MedGraphics™) and an electromagnetically braked cycle ergometer (Lode, Groningen, The Netherlands) in accordance with UK national guidelines for CPET (Levett et al., 2018). Breath‐by‐breath measurements of gas exchange were obtained using a mouthpiece connected to a MedGraphics preVent™ pneumotach device with a nose‐clip to measure both inspired and expired oxygen and carbon dioxide levels and respiratory flow. Following 3 min of resting data collection, the subject cycled at 60 rpm for 3 min in an unloaded “‘freewheeling” state. A progressively ramped period of exercise at W.min‐1 based on her stature, age, and predicted peak oxygen uptake ($\dot{V}$O_2_ peak) was then undertaken to symptom limited termination and followed by 1‐ to 5‐min recovery period.

During each CPET, the following measurements were recorded:

- *Cardiovascular*. Heart rate and electrocardiogram ST segment analysis were recorded continuously.
- *Pulmonary.* Oxygen uptake, carbon dioxide output, expiratory minute ventilation, and respiratory frequency were recorded breath by breath throughout. MedGraphics BreezeSuite™ software automatically determined $\dot{V}$O_2_ peak (defined as the highest oxygen uptake during the final 30 s of exercise reported) and oxygen uptake efficiency slope (OUES). The AT was manually interpreted by a clinician using the modified V‐slope method (Whipp, Ward, & Wasserman, 1986), supported by ventilatory equivalents for oxygen (${\dot{V}}_{E}$/$\dot{V}$O_2_) and carbon dioxide (${\dot{V}}_{E}$/$\dot{V}$CO_2_) and end‐tidal partial pressures of oxygen and carbon dioxide in accordance with UK national guidelines for perioperative CPET (Levett et al., 2018). Breath‐by‐breath data were averaged using middle five of seven breaths. Pulse oximetry was recorded throughout.

### 2.5 Data interpretation

#### 2.5.1 CPET values

For comparative purposes, the CRF of a literature‐based age‐matched control would demonstrate a $\dot{V}$O_2_ peak of 22 ml O_2_·kg^−1^·min^−1^ (Wasserman, 2012). We used reference CRF threshold values for perioperative risk from the European Association for Cardiovascular Prevention and Rehabilitation (EACPR)/American Heart Association (AHA) Scientific Statement: $\dot{V}$O_2_ at AT <11 ml O_2_·kg^−1^·min^−1^, $\dot{V}$O_2_ peak <16 ml O_2_·kg^−1^·min^−1^, and ${\dot{V}}_{E}$/$\dot{V}$CO_2_ at AT > 36. Failure to reach one or more of these thresholds cumulatively increases the perioperative risk reference (Guazzi et al., 2016). We also compared changes in CRF with known test‐retest coefficients of variation (CV) associated with both biological variation and analytical variation, indicative of Critical Difference (CD). Based on previously published works, CD represents the magnitude of change required to demonstrate a meaningful physiological change (Rose et al., 2018).

#### 2.5.2 Critical difference (CD)

CRF is a dynamic metric subject to natural variation encompassing both analytical and biological components that collectively contribute to the critical difference given by (Fraser & Fogarty, 1989):

(1) $$\text{CD}=k\sqrt{{\text{CV}}_{A}^{2}+{\text{CV}}_{B}^{2}}$$

where: *k* = constant equal to 2.77 at *p* < .05. CV_A_ = coefficient of analytical variation. CV_B_ = coefficient of biological variation.

Natural variation is described by the magnitude of CD and determines the difference in CRF required to demonstrate change not simply due to the “noise” associated with analytical imprecision (represented by CV_A_) and biological variation (represented by CV_B_), in order to determine if any change is to be considered “clinically meaningful.” We have previously calculated the CD for VO_2_‐AT, VO_2_peak, and V_E_/VCO_2_‐AT to be 19%, 13%, and 10%, respectively (Rose et al., 2018). Changes in the observed CRF metrics were retrospectively compared against these values to provide clearer insight into the true physiological benefit conferred by the respective exercise interventions.

## 3 RESULTS

Despite good self‐reported exercise capacity, an initial baseline CPET conducted 9 months after her failed esophagectomy demonstrated poor CRF, achieving peak work 73 W, AT 7.8 ml O_2_·kg^−1^·min^−1^, $\dot{V}$O_2_ peak 14.7 ml O_2_·kg^−1^·min^−1^, and ${\dot{V}}_{E}$/$\dot{V}$ CO_2_‐AT 28 (Table 1). This CPET performance was similar to that achieved prior to her original esophagectomy (Table 1).

**TABLE 1:** Cardiopulmonary exercise test results during unsupervised, moderate intensity steady‐state (MISS) training

	Pre‐esophagectomy	Pre‐MISS baseline (9 months post‐esophagectomy)	8‐week MISS completed	16‐week MISS completed	% change required based on CD	% change observed from baseline to 16 weeks
$\dot{V}$O_2_‐AT (ml O_2_·kg^−1^·min^−1^)	8.4	7.8	7.7	8.3	19%	+6%
$\dot{V}$O_2_ peak (ml O_2_·kg^−1^·min^−1^)	13.1	14.7	16.2	13.7	13%	−7%
Power at $\dot{V}$O_2_ peak (Watts)	91	73	77	70		−4%
${\dot{V}}_{E}$ (L·min^−1^)	41.3	38.0	47.8	35.8		−6%
${\dot{V}}_{E}$/$\dot{V}$CO_2_‐AT	32	28	31	30	10%	+7%
RER at peak	1.27	1.44	1.47	1.38	15%	−4%
Heart rate peak (b·min^−1^)	125	131	125	100		−24%
OUES	1,110	858	986	923	12%	+8%
Power at AT (Watts)	50	39	36	40		+3%

Abbreviations: ${\dot{V}}_{E}$, peak minute ventilation; ${\dot{V}}_{E}$/$\dot{V}$CO_2_‐AT, ventilatory equivalent for oxygen at AT; AT, anaerobic threshold; CD, critical difference; $\dot{V}$O_2_ peak, peak oxygen uptake; OUES, oxygen uptake efficiency slope; RER, respiratory exchange ratio.

A second CPET, 8 weeks later after self‐directed, unsupervised home training on a treadmill, demonstrated minimal change in AT (7.8–7.7 ml O_2_·kg^−1^·min^−1^; −1%), but small improvements in $\dot{V}$O_2_ peak (14.7–16.2 ml O_2_·kg^−1^·min^−1^; +10%), and minute ventilation (38.0–47.8 L·min^−1^; +26%) (Table 1). Following further training with a home fitness video, a third CPET 16 weeks later, demonstrated worsening of her exercise capacity (Table 1).

The 10‐week, supervised, HIIT program was well tolerated with no adverse events identified. She completed 29 of the prescribed 30 sessions (one training session was not completed due to illness). Her hemoglobin levels were normal throughout the training programs and her body mass remained constant.

**FIGURE 1:**
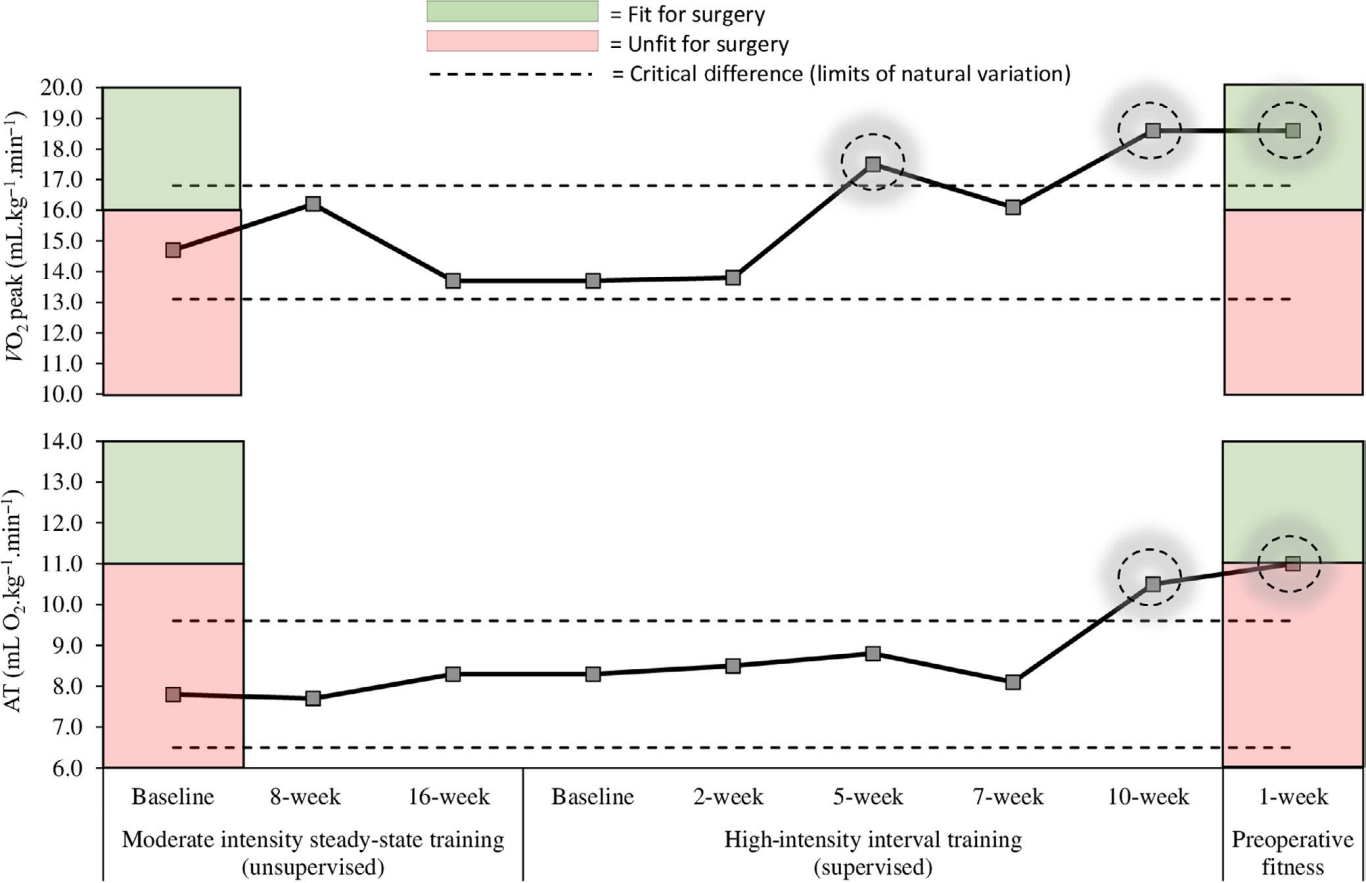
Cardiorespiratory fitness at baseline, during moderate intensity steady state (MISS) and high intensity interval training (HIIT) approaches. Following 10 weeks of HIIT (three sessions per week), fitness was maintained until the time of surgery by completion of a further two HIIT sessions per week. For comparative purposes, a literature‐based age‐matched control would demonstrate a $\dot{V}$O_2_ peak of 22 ml O_2_·kg^−1^·min^−1^ (Wasserman, 2012). AT, anaerobic threshold; $\dot{V}$O_2_ peak, peak oxygen uptake

HIIT resulted in increases in $\dot{V}$O_2_ peak (13.7–18.6 ml O_2_·kg^−1^·min^−1^; +36%), AT (8.3–10.5 ml O_2_·kg^−1^·min^−1^; +27%), peak power (70–102 W; +46%), minute ventilation (35.8–57.7 L·min^−1^; +61%), and oxygen uptake efficiency slope (OUES) (923–1,079; +17%). ${\dot{V}}_{E}$/$\dot{V}$CO_2_‐AT decreased (30–28; −7%). Peak heart rate increased 33% (100–133 b.min^−1^). (Table 2, Figure 1).

**TABLE 2:** Cardiopulmonary exercise test results during supervised high‐intensity interval training (HIIT)

	Pre‐HIIT baseline	2‐week HIIT completed	5‐week HIIT completed	7‐week HIIT completed	10‐weeks HIIT completed	% change required based on CD	% change from baseline to 10 weeks
$\dot{V}$O_2_‐AT (ml O_2_·kg^−1^·min^−1^)	8.3	8.5	8.8	8.1	10.5	19%	+27%
$\dot{V}$O_2_ peak (ml O_2_·kg^−1^·min^−1^)	13.7	13.8	17.5	16.1	18.6	13%	+36%
Power at $\dot{V}$O_2_ peak (Watts)	70	76	91	95	102		+46%
${\dot{V}}_{E}$ (L·min^−1^)	35.8	32.7	49.1	51.4	57.7		+61%
${\dot{V}}_{E}$/$\dot{V}$CO_2_‐AT	30	28	30	28	28	10%	−7%
RER at peak	1.38	1.42	1.38	1.60	1.55	15%	+12%
Heart rate peak (b·min^−1^)	100	120	118	120	133		+33%
OUES	923	969	1,000	944	1,079	12%	+17%
Power at AT (Watts)	40	36	40	40	52		+30%

Abbreviations: ${\dot{V}}_{E}$, peak minute ventilation; ${\dot{V}}_{E}$/$\dot{V}$CO_2_‐AT, ventilatory equivalent for oxygen at AT; AT, anaerobic threshold; CD, critical difference; $\dot{V}$O_2_ peak, peak oxygen uptake; OUES, oxygen uptake efficiency slope; RER, respiratory exchange ratio.

## 4 DISCUSSION

The patient's CRF at baseline was considerably lower than literature‐based age‐matched controls and also confirmed a high level of risk for major surgery when compared with reference CRF threshold values for perioperative risk stratification. Supervised HIIT training enabled impressive improvement in excess of natural variation and was clinically significant based on application of the critical difference. Our patient's improved CRF as demonstrated by her CPET metrics resulted in the reclassification of her risk for major surgery into a low risk group. This clinical case study highlights that HIIT in the high‐risk patient preparing for major intra‐abdominal surgery is effective. HIIT improvements in CRF were incurred over a short period of time and were considered clinically meaningful enabling the patient to transcend the “fitness” boundary ahead of major surgery. Collectively, our findings support the implementation of HIIT as an effective prehabilitation strategy with the potential to optimize perioperative outcome.

The “high‐risk surgical patient” accounts for 13% of cases in the United Kingdom, but contributes to over 80% postoperative deaths and complications (Pearse et al., 2006). The principle of prehabilitation is to improve cardiovascular, respiratory, and muscular conditioning and can be considered analogous to the preparation of an individual for a marathon event (Wynter‐Blyth & Moorthy, 2017). Improving a patient's physiological reserve allows them to meet the demands of this perioperative stress, reducing the risk of complications and death. A multimodal prehabilitation program allows other factors such as smoking, alcohol, nutrition, and anemia to be addressed (Tew et al., 2018). The optimum components of an exercise program have yet to be elucidated as much of the evidence is relatively recent (Minnella & Carli, 2018). Given the short time period that cancer patients have between diagnosis and surgery, HIIT training seems to confer the greatest advantages and current trials are ongoing to determine this (Woodfield et al., 2018).

We acknowledge the idiosyncrasies present when comparing the type of exercise intervention (supervised vs. unsupervised), intensity of exercise (HIIT vs. MISS), and mode of exercise (walking vs. cycling), and that a controlled experiment with an age‐matched healthy participant was outside the scope of this work. We simply aimed to demonstrate the impact of a theoretically effective exercise intervention on a single patient to improve clinical outcome. The efficacy of our HIIT intervention may be attributed to some key factors. First, the HIIT program was individualized using the cycle ergometer to adjust work rate based on the patient's power output at two measured physiological parameters (AT and $\dot{V}$O_2_ peak). This allowed targeted training using a planned program of exercise. Second, the HIIT program was supervised throughout by both a medical professional and exercise physiologist. This joint supervision allowed for psychological, behavioral, and environmental factors to be addressed through regular encouragement, reassurance, and motivation in a safe and secure environment. While the patient ultimately must do the training, the health professionals must supervise the HIIT program to harness and maintain patient motivation while ensuring safety. While we have demonstrated beneficial increases in CRF, the present HIIT program is admittedly resource intensive in terms of equipment and professional input. Further research is required to evaluate the potential for its widespread implementation in the preoperative setting, given the inevitable financial and logistical constraints.

Studies in healthy participants and patients with established cardiometabolic disease have consistently demonstrated a greater increase in maximal oxygen consumption ($\dot{V}$O_2_ max) following HIIT compared to MISS (Milanovic, Sporis, & Weston, 2015). The associated $\dot{V}$O_2_ max increase is associated with elevated peroxisome proliferator‐activated receptor gamma coactivator 1‐alpha mRNA (Gibala et al., 2009), a moderator of skeletal muscle mitochondrial biogenesis which sits “front and central” in terms of the primary mechanism underpinning its superior cardiopulmonary adaptive benefits. Furthermore, an increase in citrate synthase (a marker of muscle oxidative capacity) has also been reported (Burgomaster, Hughes, Heigenhauser, Bradwell, & Gibala, 2005). Systemic vascular function has also been shown to improve following HIIT (Molmen‐Hansen et al., 2012), the likely consequence of an “optimized” blood flow‐shear phenotype, triggering calcium influx into the hyperpolarized endothelial cells (Cooke, Rossitch, Andon, Loscalzo, & Dzau, 1991) upregulating endothelial nitric oxide synthase (Bolduc, Thorin‐Trescases, & Thorin, 2013). Collectively, these studies demonstrate that despite shorter bouts of activity, albeit at higher intensity, HIIT has the capacity to further potentiate physiological adaptation compared to MISS, which lies at the heart of its current popularity. Indeed, we demonstrated that the majority of adaptive benefit was incurred within the first 5 weeks of HIIT, suggesting that training interventions as short as this may prove “sufficient” allowing the patient to transcend the “fitness for surgery” boundary.

In conclusion, HIIT was shown to be a feasible, safe, and well‐tolerated exercise intervention that was associated with impressive improvements in CRF enabling a single patient to be classified as “fit” for major surgery. Collectively, our findings support the detailed investigation of HIIT as an effective prehabilitation strategy with the potential to optimize perioperative outcome.

## CONFLICT OF INTEREST

No conflicts of interest (financial or otherwise) are declared by the authors.

## AUTHOR CONTRIBUTIONS

R.D., I.A., and G.R. performed the experiment and supervised the exercise interventions. M.A., R.D., I.A., G.R., and D.M.B. analyzed the data and interpreted the findings. M.A., G.R., R.D., and I.A. drafted the manuscript. All authors edited and revised the manuscript and approved the final version.

## Data Availability

The data that support the findings of this study are available on request from the corresponding author. The data are not publicly available due to privacy or ethical restrictions.
